# Dust Under the Radar: Rethinking How to Evaluate the Impacts of Dust Events on Air Quality in the United States

**DOI:** 10.1029/2023GH000953

**Published:** 2023-12-08

**Authors:** K. Ardon‐Dryer, K. R. Clifford, J. L. Hand

**Affiliations:** ^1^ Department of Geosciences Texas Tech University Lubbock TX USA; ^2^ Western Water Assessment & Department of Geography University of Colorado Boulder CO USA; ^3^ Cooperative Institute for Research in the Atmosphere (CIRA) Colorado State University Fort Collins CO USA

**Keywords:** dust events, dust storm, air quality, PM10, PM2.5

## Abstract

Dust is an important and complex constituent of the atmospheric system, having significant impacts on the environment, climate, air quality, and human health. Although dust events are common across many regions of the United States, their impacts are not often prioritized in air quality mitigation strategies. We argue that there are at least three factors that result in underestimation of the social and environmental impact of dust events, making them receive less attention. These include (a) sparse monitoring stations with irregular spatial distribution in dust‐influenced regions, (b) inconsistency with dust sampling methods, and (c) sampling frequency and schedules, which can lead to missed dust events or underestimation of dust particle concentrations. Without addressing these three factors, it is challenging to characterize and understand the full air quality impacts of dust events in the United States. This paper highlights the need for additional monitoring to measure these events so that we can more fully evaluate and understand their impacts, as they are predicted to increase with climate change.

## Key Points

- Different factors result in an underestimation of the social and environmental impact of dust events, making them receive less attention
- There is a lack of consistency in dust definitions and sampling methods
- Sampling frequency and schedules of dust events can lead to missed dust events or underestimation of dust particle concentrations

## 1 Introduction

Dust particles are emitted and suspended in the atmosphere through entrainment by strong winds across erodible surfaces. This process results in the creation of dust events known as blowing dust events or dust storms; the latter is considered more severe and is defined by conditions resulting in horizontal visibility below 1 km, while blowing dust events are defined by visibility conditions from 1 to 10 km (WMO, 2019). These dust events have an important effect on the atmospheric system, influencing radiation (Lau et al., 2020), cloud formation (Chen et al., 2019), and the atmospheric vertical electric field (Ardon‐Dryer, Chmielewski, et al., 2022). Air quality, human well‐being, and human health are also affected, mainly negatively (Ardon‐Dryer & Kelley, 2022; Tong et al., 2023a, 2023b). Dust in North America is estimated to contribute ∼2.5% (0.3–0.9 Tg) of the global dust loading for particles with diameters up to 20 μm (PM_20_) (Kok et al., 2021), yet some studies estimated much higher concentrations as found by Urban et al. (2018), who estimated higher dust emissions just from the Mojave Desert (7–15 Tg year^−1^ for PM_20_). Further, climate models predict that with increases in aridity, dust events will likely increase in the future (Achakulwisut et al., 2018; Brey et al., 2020; Pu & Ginoux, 2017).

Besides monitoring, other challenges exist when characterizing and managing dust levels in the atmosphere. At the most basic level, a lack of a consistent, or shared, definition of dust amongst scientists, the public, and regulators (Kroepsch & Clifford, 2022; Tong et al., 2022) limits the advancement of dust science, in part because of a lack of consensus regarding standard dust measurements. For example, airborne dust is defined in different ways. Some studies define dust based on the concentration of PM_10_ (mass of particles with diameters less than 10 μm; Lei & Wang, 2014; Eagar et al., 2017). Other studies have used coarse mass (PM_10_ − PM_2.5_) (where PM_2.5_ is the mass of particles with diameters less than 2.5 μm; Hand et al., 2017), or the ratio of PM_2.5_ and PM_10_ (Tong et al., 2017) to indicate dust impacts. Fine dust, based on elemental composition, has also been used to characterize dust impacts (e.g., Chow et al., 2015; Hand et al., 2017; Liu et al., 2022; Malm et al., 1994). The definition of the severity of the dust impact is another challenge, as “blowing dust event” and “dust storm” are often used interchangeably or broadly, which can cause confusion or limit the interpretation of the impacts of dust events, especially with respect to health applications (Ardon‐Dryer et al., 2023). Another challenge is that dust is often dismissed in policy and management decisions (Clifford, 2022). For example, a rule within the Clean Air Act allows dust events to be removed from regulatory data sets when considered an “exceptional” event (Clifford, 2021). Such data exclusions occur in both rural and urban settings, with fine and coarse particles, and while more common in the arid West, are a potential issue across the country. These challenges increase the difficulty of accurately tracking spatial and temporal trends in dust and its impacts on air quality.

We highlight three major factors that interfere with accurately estimating the impacts of dust events on air quality. These issues likely lead to a gap in knowledge of dust concentration, composition, and size‐composition relationships, which limits our understanding of its spatial and temporal air quality impacts and other issues associated with and caused by dust across the United States. These three factors are described below.

## 2 Large Spatial Gaps in Data Result in Unmonitored Events

**Figure 1:**
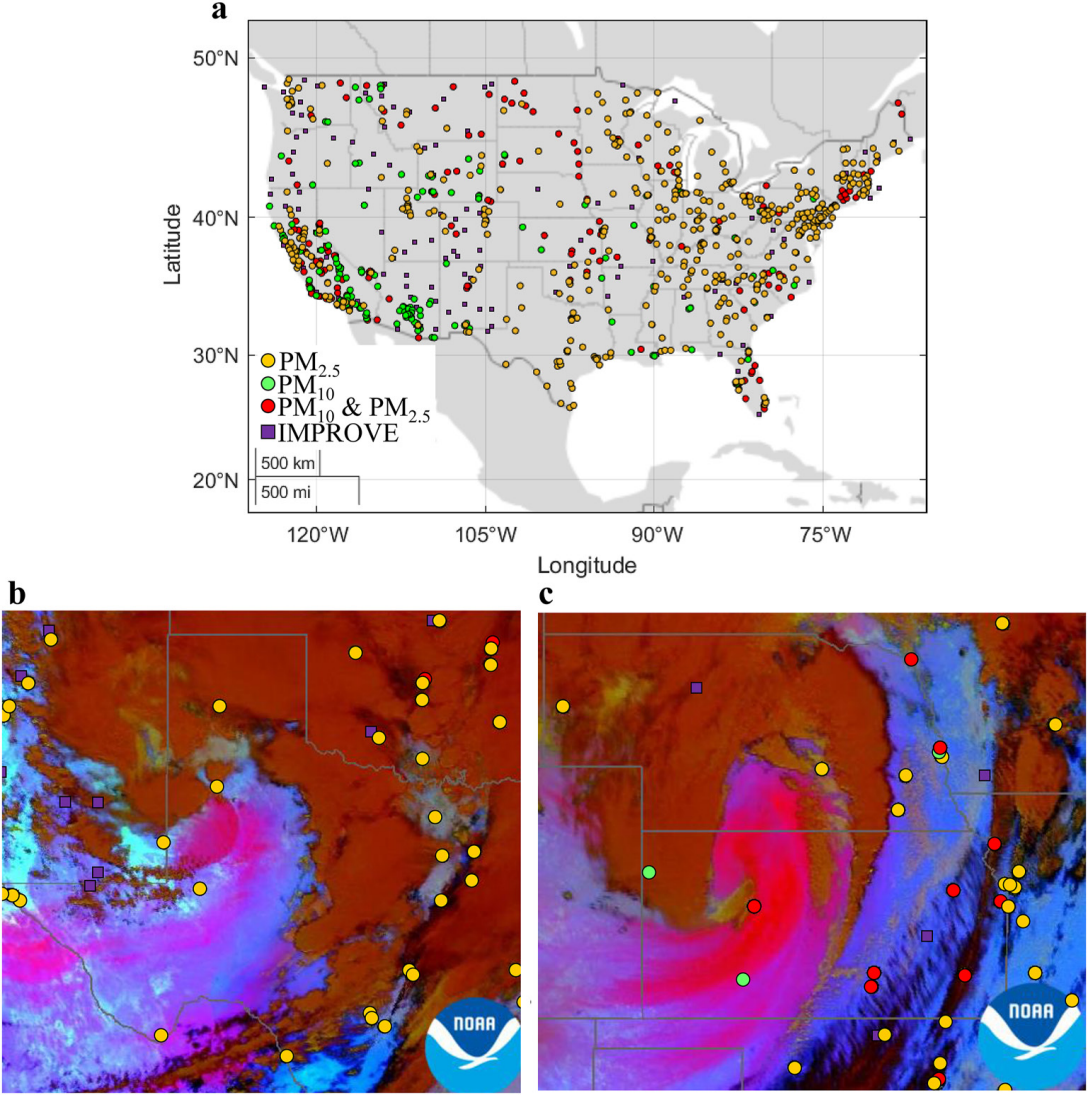
(a) Large‐scale monitoring networks, including Environmental Protection Agency's federal reference method networks for only PM_2.5_ (orange), only PM_10_ (green), collocated PM_2.5_ and PM_10_ (red), as well as Interagency Monitoring of Protected Visual Environments (IMPROVE) sites (PM_2.5_ and PM_10_, purple) that were active in 2020. (b) Examples of dust events (in pink; based on dust RGB product that contrast dust particles from clouds using channel differencing and the infrared thermal channel; Dust RGB, 2023) captured by the GOES‐16 satellite over Texas (22 March 2021), and (c) over Kansas (15 December 2021), highlighting areas void of sensors, with PM_2.5_ (orange), PM_10_ (green), PM_2.5_ with PM_10_ (red), and IMPROVE sites (purple). Satellite images retrieved from AerosolWatch (https://www.star.nesdis.noaa.gov/smcd/spb/aq/AerosolWatch/, accessed on 11 February 2023).

Dust data are often based on the impact of dust on particulate matter (PM) measurements of PM_2.5_ and PM_10_ gravimetric mass, which also include contributions from other aerosol species. PM_2.5_ and PM_10_ are routinely monitored by the United States Environmental Protection Agency (EPA) as part of large‐scale monitoring networks in mostly urban settings in support of the National Ambient Air Quality Standards (NAAQS). In addition, the EPA operates the large‐scale Chemical Speciation Network (CSN) for health exposure studies (Solomon et al., 2014), with sites mainly in urban and suburban settings. Aerosol speciation measurements also occur at remote and rural sites by the Interagency Monitoring of Protected Visual Environments (IMPROVE) aerosol speciation network for the purpose of monitoring visibility (Malm et al., 1994; see Figure 1a for map of network site locations). While the networks often have similar measurement strategies and sampling schedules, they were not initially designed as dust monitoring networks. However, combining data from several hundred sites across the networks has led to a better understanding of large‐scale spatial patterns and seasonality of dust across the United States (Aryal & Evans, 2022; Hand et al., 2017, 2019; Tong et al., 2017). Nevertheless, it is clear from these studies that the spatial gaps between and within monitoring networks are limiting our knowledge of dust impacts on air quality.

In particular, regions influenced by dust events, as observed from satellites, often do not have PM monitors. For example, only 21% of counties in the United States have PM_2.5_ monitors (Sullivan & Krupnick, 2018), and most of the PM monitoring that supports NAAQS and other health exposure studies are in areas with high population density, resulting in spatial gaps in many rural areas that often experience dust events (see examples of dust events in Figures 1b and 1c, highlighting the large area that was impacted by dust, areas of ∼55,000 km^2^ and ∼85,000 km^2^ respectively, that are not captured by any sensors). In recent years, commercially available, low‐cost air quality sensors have become common, and regulatory agencies started using them to obtain more granular information on air quality spatial and temporal distribution (Jaffe et al., 2023). However, the accuracy and precision of these sensors need to be characterized (Zheng et al., 2018), the sensors require calibrations (Ardon‐Dryer et al., 2020) and recent work suggests they are unable to accurately characterize coarse particles (>2.5 μm) (Jaffe et al., 2023; Kaur & Kelly, 2023; Rueda et al., 2023) and they still contain spatial gaps. These spatial gaps limit our ability to fully quantify the number and nature of dust events and their subsequent impacts. While satellite observations provide useful spatial and temporal information regarding large dust events, as well as identify affected regions void of monitors, satellites cannot replace ground‐based monitoring to adequately characterize dust impacts on surface air quality, and satellites may miss dust events depending on the timing of satellite coverage.

## 3 Monitors May Miss Dust Events Due To Particle Size

**Figure 2:**
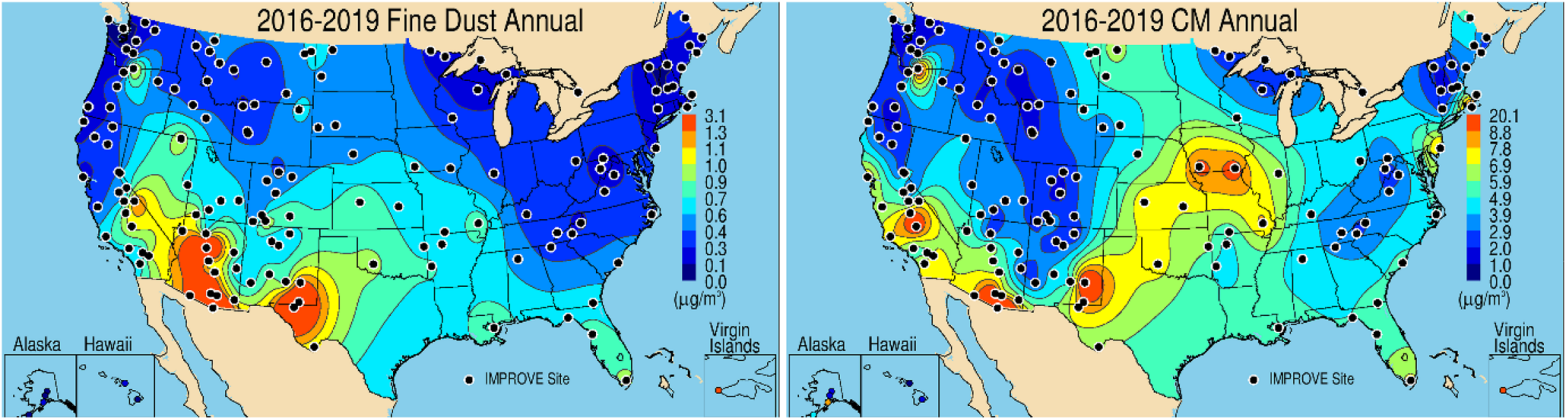
2016–2019 Interagency Monitoring of Protected Visual Environments annual mean (a) reconstructed fine (PM_2.5_) dust (μg m^−3^) and (b) gravimetric coarse mass (PM_10_ − PM_2.5_, μg m^−3^), adapted from Hand et al., 2023.

Dust particle size can range across several orders of magnitude (∼100 nm–100 μm, Ardon‐Dryer, Kelley, et al., 2022; Neff et al., 2013; Scheuvens & Kandler, 2014). Some of these inhalable particles have serious health effects (Martinelli et al., 2013; Tobias et al., 2019). Measurements of PM_2.5_ or dust concentrations derived from PM_2.5_ elemental speciation measurements could lead to underestimates of dust impacts on air quality as they miss the fraction of dust particles which are often associated with the coarse aerosol mode (PM_10_–PM_2.5_), Total Suspended Particles (TSP; Neff et al., 2013; Reynolds et al., 2016) or even just of giant mode dust (van der Does et al., 2018). Differences in spatial and seasonal variability of reconstructed fine dust and coarse mass suggest that knowledge of dust size distribution and coarse mass composition may be critical for understanding and reconciling PM_2.5_ and PM_10_ data that are influenced by dust events (Hand et al., 2023). Annual mean fine dust concentrations from 2016 through 2019 at sites from the IMPROVE network are shown in Figure 2a, compared to the annual mean coarse mass at the same sites (Figure 2b). Differences in spatial patterns, especially at sites in the central United States, may be due to dust size distribution, or different sources and composition of coarse mass (Bondy et al., 2018; Malm et al., 2007). While IMPROVE provides chemicals speciated for PM_2.5_, no speciation information is available for PM_10_. Without these additional measurements, we can only speculate regarding the sources and transport of dust. In addition, biases in fine dust concentrations derived from measurements using collocated but different samplers from the CSN and IMPROVE networks are likely due to the sharpness of the cut point of the sampler that allows varying amounts of coarse mode dust to be collected on the PM_2.5_ filter (Gorham et al., 2021; Hand et al., 2012, 2023; Solomon et al., 2014).

## 4 Sampling Frequency and Duration Limit the Ability to Detect the Impact of Dust Events on Air Quality

**Figure 3:**
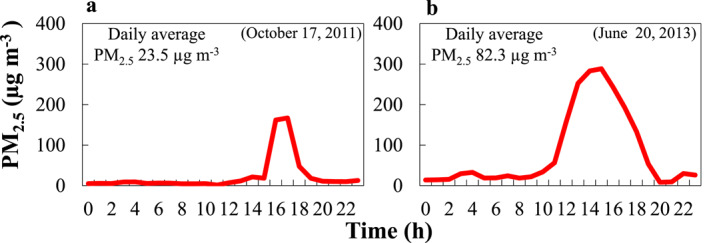
Hourly gravimetric PM_2.5_ measurements of dust events measured by the Texas Commission on Environmental Quality from Lubbock, Texas with daily values (a) Example of short‐term dust events that do not exceed the daily PM_2.5_ threshold. (b) Example of the hourly values during longer‐term dust events show high daily PM_2.5_ values.

While some of the EPA federal reference method PM_2.5_ and PM_10_ sensors provide hourly values (EPA, 2020), most networks collect aerosol on filters for 24 hr every third, sixth, or twelfth day, depending on the site. Given these schedules, dust events are often missed. Dust events can happen on time scales of hours or less, and some may exceed EPA daily thresholds, which are 35 μg m^−3^ for PM_2.5_ and 150 μg m^−3^ for PM_10_ (Figure 3). Integrated samples or 24‐hr averages of hourly data may mask the severity and true impact of these events, leading to a low daily threshold (Figure 3a). In a recent study, Ardon‐Dryer and Kelley (2022) showed that even hourly PM measurements may mask the contribution of PM concentrations during short‐duration (sub‐hourly) dust events, leading to underestimation of the PM concentrations during these short and intense events. While overlooked, these short‐duration dust events still carry important impacts; studies found that exposure to high PM concentrations (of fine and coarse PM), can penetrate the tracheobronchial and alveolar region (Kodros et al., 2018), causing health issues including cardiac, cerebrovascular and respiratory diseases and mortality (Alessandrini et al., 2013; Malig & Ostro, 2009; Mallone et al., 2011; Martinelli et al., 2013; Pérez et al., 2008). Yet, it is still unclear what the health consequences are of such intense but relatively short exposure to dust particles (e.g., acute exposure). This raises questions about whether we might need different air quality standards that account for such short‐duration dust emissions (Bouet et al., 2019), especially as many of the dust events in the United States last for an hour or less (Ardon‐Dryer et al., 2023).

## 5 Moving Forward

Dust can contribute significantly to PM in the United States, especially on a seasonal basis. Current estimates suggest that half of the PM_2.5_ mass during spring in the Southwest is fine dust, and coarse mass can contribute more than 70% to PM_10_ across the West on an annual basis (Hand et al., 2017, 2019, 2023). Others indicate on high presence of large particles as measured by the TSP sample (Neff et al., 2013; Reynolds et al., 2016). However, without consistent, more frequent measurements in dust‐influenced regions, these are likely underestimates. Recent studies suggest that dust loading will increase due to climate change (Achakulwisut et al., 2018; Pu & Ginoux, 2017) but without additional data, we are limited in our ability to fully characterize its impacts on air quality and therefore on health. Even designing appropriate mitigation strategies to reduce its future impacts will require accurate characterizations of dust events. The first step is to enhance the monitoring network with additional monitors that sample more frequently, especially in dust‐influenced regions. Consistent measurements of particle size and composition would also help characterize the environmental, climate, and human health impacts of dust events. Because these additions are likely cost prohibitive, the development and deployment of low‐cost sensors that can accurately measure coarse PM concentrations during dust events may be a path forward to help understand the true impacts of dust on air quality in the United States. These dust‐effective low‐cost sensors could potentially be placed across urban and rural locations, or in existing networks (e.g., Mesonet stations, National Wind Erosion Research Network, IMPROVE, National Atmospheric Deposition Program and others; USGS, 2023; Webb et al., 2016; West Texas Mesonet, 2023), ensuring their information will be distributed equitably.

## Conflict of Interest

The authors declare no conflicts of interest relevant to this study.

## Data Availability

Hourly gravimetric PM_2.5_ measurements were used from the Texas Commission on Environmental Quality (2023) Information from IMPROVE network, a collaborative association of state, tribal, and federal agencies, and international partners funded by the U.S. Environmental Protection Agency (EPA) with research support from the National Park Service, were retrieved from IMPROVE (2023). Satellite images retrieved from AerosolWatch website (AerosolWatch, 2023).
